# Survivorship Struggles: Navigating Etiologies and Clinical Parameters of Febrile Neutropenia During Induction Chemotherapy in Acute Leukemia Patients

**DOI:** 10.7759/cureus.54935

**Published:** 2024-02-26

**Authors:** Muhammad Haris Khan, Muhammad Adnan Wattoo, Habib ur Rehman Butt, Muhammad Zaid, Umair Tahir, Mehwish Kaneez, Usaid Munir Joyia, Syed Faheem Bukhari

**Affiliations:** 1 Internal Medicine, Gujranwala Medical College Teaching Hospital, Gujranwala, PAK; 2 Internal Medicine, Jinnah Hospital, Lahore, PAK; 3 Internal Medicine, Islam Medical College, Sialkot, PAK; 4 Internal Medicine, Avicenna Medical and Dental College, Lahore, PAK; 5 Internal Medicine, Sheffield Teaching Hospitals NHS Foundation Trust, Sheffield, GBR; 6 Internal Medicine, Rawalpindi Medical University, Rawalpindi, PAK; 7 Infectious Diseases, Rawalpindi Medical University, Rawalpindi, PAK

**Keywords:** acute leukemia, antimicrobial resistance, infection-associated mortality, induction chemotherapy, febrile neutropenia

## Abstract

Background

Acute leukemia, characterized by the uncontrolled proliferation of immature white blood cell precursors, poses significant challenges during induction chemotherapy, including the elevated risk of febrile neutropenia and its associated complications. Our study aims to explain the clinical and etiological parameters of these patients in a resource-limited setting.

Methods

This retrospective study focused on a total of 102 adult patients with acute leukemia who developed febrile neutropenia during the induction chemotherapy phase. Patients with disease relapse, prior bone marrow transplantation, and cases of acute promyelocytic leukemia were excluded from the study. Demographical characteristics, symptoms at presentation, diagnoses, infectious causes, and outcomes were systematically reported. Infectious etiologies and detailed culture reports were meticulously tabulated, and subsequent data were analyzed.

Results

Of the 102 patients, 43 (42.2%) were males, with a mean age of 31.9 ± 6.5 years. During the induction chemotherapy, a total of 31 patients died of complicated febrile neutropenia. Severe vomiting was the most common symptom present in 37 (36.2%), followed by cough in 35 (34.3%) and loose stools in 28 (27.5%). Community-acquired pneumonia, neutropenic sepsis, and neutropenic colitis were among the most common etiologies of febrile neutropenia. A total of 72 (70.6%) patients had culture-proven multidrug-resistant Gram-negative bacteremia that contributed to poor outcomes.

Conclusions

Acute leukemia patients undergoing induction chemotherapy face high infection-associated mortality due to their immunocompromised state. Inadequate infection control measures and antimicrobial resistance contribute to the emergence of multidrug-resistant organisms. Enhanced infection prevention strategies and evidence-based antibiotic prescription guidelines are need of time in resource-limited settings such as Pakistan to address febrile neutropenia complications and bridge the existing care gap in its management.

## Introduction

Acute leukemia comprises a group of hematologic malignancies marked by the uncontrolled proliferation and infiltration of immature precursors of white blood cells [1]. This uncontrolled growth results in profound thrombocytopenia, anemia, and leukopenia, ultimately progressing to a fatal outcome if left untreated [2]. Induction chemotherapy is a cornerstone in the initiation of treatment of acute leukemias as it plays a pivotal role in achieving early disease remission [3]. However, the path to remission is fraught with complications including tumor lysis syndrome, renal failure, immunosuppression, and febrile neutropenia [4]. During induction chemotherapy, the increased vulnerability to infections due to low neutrophil counts or neutropenia becomes a formidable hurdle [5]. Statistics show that nearly 80% of patients with hematological malignancies experience febrile neutropenia [6]. It not only exacerbates the morbidity but also poses a substantial management challenge, particularly in resource-constrained settings such as Pakistan.

Numerous studies have investigated the outcomes of febrile neutropenia in developed countries; however, limited data are available on the topic in developing countries [1,6,7]. Therefore, this research article aims to address these knowledge gaps and analyze the clinical and microbiological spectrum of infections that cause febrile neutropenia in such patients. Furthermore, the article aims to shed light on the significant implications of febrile neutropenia in the context of a developing country, emphasizing the unique challenges faced in managing infections during induction chemotherapy. Finally, the study also aims to explore the profound impact of these infections on altering the disease course and contributing to survivorship.

## Materials and methods

This retrospective cross-sectional study was conducted in the Department of Internal Medicine, Rawalpindi Medical University, and Allied Hospitals, Rawalpindi, from January 2019 to May 2022. The study included adult patients diagnosed with acute myeloid leukemia (AML) or acute lymphoblastic leukemia (ALL) who developed febrile neutropenia during induction chemotherapy. The diagnosis of acute leukemia was made on bone marrow morphology, cytology, flow cytometry, and immunophenotyping. Pediatric patients with ages below 18 years, relapsed disease, history of prior bone marrow transplantation, evidence of infection before the start of chemotherapy, and cases of acute promyelocytic leukemia (APML) were excluded from the study. Moreover, patients with febrile neutropenia with no proven infection were also excluded from the analysis. The ethical review board of Rawalpindi Medical University granted ethical approval for the study (approval number 2022-MED-14773).

All patients with a confirmed diagnosis of ALL and AML were admitted for 48-72 hours for the management of tumor lysis syndrome, followed by induction chemotherapy. Patients were subsequently discharged if clinically well after day five of the initiation of chemotherapy. The remaining days of the induction chemotherapy was administered as outpatient and patients were closely followed with twice weekly baseline investigations. During induction chemotherapy, patients were advised to seek immediate medical attention if they developed a fever. A single temperature spike of 38.3°C or 101°F was defined as fever [1,6]. Upon presentation to the emergency department, febrile patients underwent a thorough assessment, including a detailed history and physical examination. Essential diagnostic tests, such as complete blood count, serum electrolytes, blood cultures, urine culture, or chest imaging (X-ray, CT scans), were performed as required. Additionally, a broad-spectrum antibiotic treatment (piperacillin and tazobactam) was promptly injected. Patients with an absolute neutrophil count below 1,000 were clinically presumed to have an infection and were admitted to the hospital. Infection validation relied on microbiological evidence from blood cultures, urine cultures, tissue specimens, fine needle aspirations, or endoscopically obtained core biopsy of sinuses. Blood cultures were initially obtained upon admission and repeated as necessary, particularly in cases of new fever, changes in antibiotic therapy, or persistent fever.

Data on patient demographics, culture reports, infectious causes, diagnoses, and outcomes were gathered from computerized records and patient files. Descriptive statistics, including mean and standard deviation for numerical data and frequencies and percentages for categorical data, were utilized to summarize the data. Subsequently, the analysis of all gathered data was performed utilizing Statistical Product and Service Solutions (Statistical Product and Service Solutions (SPSS) (IBM SPSS Statistics for Windows, Armonk, NY).

## Results

In the present analysis of 102 patients, the mean age was 31.9 ± 6.5 years, with a range between 21-52 years. The majority of the patients were 31-40 years of age. The baseline characteristics of study participants before treatment are delineated in Table 1.

**Table 1:** Baseline characteristics of study participants before treatment.

Parameter		Frequency	Percentages
Gender	Male	43	42.2%
	Female	59	57.8%
Smoking status	Smoker	18	17.6%
	Nonsmoker	84	82.4%
Type of leukemia	Acute lymphoblastic leukemia	81	79.4%
	Acute myeloid leukemia	21	20.6%
Age	21-30	35	34.4%
	31-40	38	37.2%
	41-50	24	23.5%
	Above 50	5	4.9%
Baseline tumor lysis profile	Deranged	9	8.8%
	Normal	93	91.2%
Risk stratification	Low	62	60.8%
	Intermediate	28	27.4%
	High	12	11.8%

A total of 31 (30.4%) patients died because of various infection-related complications. Severe vomiting, cough, loose stools, and pain were the most common presenting symptoms. A breakdown of various clinical parameters in our study population is elucidated in Table 2.

**Table 2:** Clinical parameters of patients presenting with febrile neutropenia.

Parameter		Frequency	Percentages
Presenting complaints other than fever	Cough	35	34.3%
	Loose stools	28	27.5%
	Seizures	2	2%
	Severe vomiting	37	36.2%
	Pain	23	22.5%
Reason for febrile neutropenia	Community-acquired pneumonia	27	26.5
	Hospital-acquired pneumonia	13	12.7%
	Meningitis	2	2%
	Neutropenic colitis	21	20.6%
	Biopsy site infection	3	2.9%
	Neutropenic sepsis	22	21.6%
	Mucosal barrier injury	3	2.9%
	Urinary tract infection	9	8.8%
	Cellulitis	1	1%
	Tuberculosis	1	1%
Mortality status	Dead	31	30.4%
	Alive	71	69.6%

A majority of isolated microorganisms were predominantly multidrug-resistant gram-negative bacteria. Fungi and cytomegalovirus were also identified as causes of febrile neutropenia. An illustration of isolated organisms can be seen in Table 3.

**Table 3:** An elucidation of isolated infectious organisms in our patients.

Organism type	Microorganism isolated	Mode of isolation	Total frequency	Percentage
Gram-positive isolates	Methicillin-resistant Staphylococcus aureus	Blood culture	13	12.7%
	Vancomycin-resistant *Enterobacter*	Wound culture	3	2.9%
	Vancomycin-resistant *Staphylococcus aureus*	Blood culture	2	2%
Gram-negative isolates	Klebsiella pneumoniae	Tracheal aspirates	5	4.9%
	Klebsiella pneumoniae	Blood cultures	11	10.8%
	Multidrug-resistant* Pseudomonas aeruginosa*	Tracheal aspirates	8	7.8%
	Stenotrophomonas	Tracheal aspirates	6	5.9%
	Multidrug-resistant *Escherichia coli*	Blood cultures	13	12.8%
	Acinetobacter baumanii	Blood cultures	8	7.8%
	Pseudomonas aeruginosa	Blood and wound cultures	12	11.8%
	Multidrug-resistant *Escherichia coli*	Urine cultures	9	8.8%
Fungal	Mucor mycosis	Biopsy specimen	2	2%
	Invasive candida	Blood cultures	5	4.9%
	Cryptococcus neoformans	Cerebrospinal fluid	2	2%
Viruses	Cytomegalovirus	Blood	3	2.9%

## Discussion

Patients with acute leukemia on induction chemotherapy undergo severe myelosuppression that predisposes them to both community and hospital-acquired infections. These infections lead to adverse outcomes during the intense phase of treatment and exacerbate mortality [7]. Despite the existence of comprehensive guidelines for supportive care and management of infections, complicated febrile neutropenia remains the most prevalent cause of mortality during inducting chemotherapy in acute leukemia patients, and the results of our study are suggestive of this finding [8].

Febrile neutropenia is a life-threatening oncological emergency that emphasizes the need for comprehensive research and improved therapeutic strategies to address the challenges faced by patients undergoing induction chemotherapy [8,9]. A developing nation such as Pakistan grapples with the complexities of the management of febrile neutropenia, where limited resources and access to advanced medical care add a layer of adversity [9]. Statistics from different developing countries report high mortality in acute leukemia, ranging between 25% to 35%, which is consistent with the results of our study [9-11]. However, studies from developed countries in Europe, China, and North America report a lower mortality rate of 5% to 10% [3,6,12]. The discrepancy in mortality rates elucidates the care gap that acute leukemia patients receive across the globe in the management of febrile neutropenia. Furthermore, improved survival in developed countries also signifies the need for comprehensive infection prevention and management strategies [13]. This highlights the critical need for robust approaches to address infections holistically, emphasizing the importance of such measures in ensuring favorable outcomes [14].

Previous studies show that antimicrobial resistance and hospital-acquired infections are some of the most important causes of mortality during febrile neutropenia episodes especially during induction chemotherapy [15,16]. In our study, severe gram-negative neutropenic sepsis and fatal outcomes are frequently linked to stubborn gram-negative organisms such as Klebsiella, Pseudomonas, and Escherichia coli, which show resistance to various drugs. This pattern is consistent with findings from other studies, which report nearly 70% of the infections in complicated febrile neutropenia are hospital-acquired [9,17]. The widespread challenge posed by these multidrug-resistant gram-negative organisms in causing severe infections jeopardizes patient outcomes and increases the risk for mortality [2,11]. Similarly, the heightened prevalence of multidrug-resistant gram-positive strains also advocates this finding.

Mishandling of medical equipment (urinary catheters, central venous lines, arterial lines, gastric tubes, etc.), deficiencies in hand hygiene, and the presence of contaminated surfaces are the primary reasons for the spread of nosocomial infection and development of multidrug-resistant gram-negative organisms [18]. These deficiencies promote the spread of hospital-acquired infections, especially in immunocompromised patients leading to infection-related complications and poor outcomes [15]. These factors demand a combined effort involving various healthcare disciplines to ensure infection control measures, such as proper hand hygiene, thorough sterilization of contaminated surfaces, and rational use of antimicrobial agents [19]. Furthermore, it is crucial to conduct regular surveillance for infections, promptly diagnose them, and tailor treatment based on culture results [20]. A multidisciplinary approach of the infection control department in controlling the spread of infection and the infectious disease department for approval of broad-spectrum antibiotics can aid in decreasing the incidence of nosocomial infections [19,20]. This comprehensive approach is essential to decrease complications, mitigate antibiotic resistance, and improve survivorship.

Our study is not without limitations, including the reliance on data from a single center and utilization of a retrospective study design. The study outcomes could have been more comprehensive with a more extensive, nationwide data collection across multiple centers. Nevertheless, our research sheds light on crucial aspects of infections and outcomes of febrile neutropenia in acute leukemia. It underscores the necessity for localized guidelines in handling infections during neutropenic sepsis in these patients. Identification of effective strategies to mitigate infection risks and enhance overall survival in these patients is paramount. Moreover, our study provides meaningful insights into the problem of antimicrobial resistance and the need for antimicrobial stewardship in developing countries such as Pakistan.

## Conclusions

Acute leukemia patients on induction chemotherapy have a high infection-associated mortality because of an immunocompromised state. Poor infection control strategies and lack of antimicrobial stewardship are the main culprits of resistant microorganisms that contribute to fatal infections. The prevalence of multidrug-resistant gram-negative organisms further complicates the scenario, posing a serious threat to patient outcomes. There is an urgent need for comprehensive infection prevention strategies and careful antibiotic prescription guidelines especially in resource-constrained settings such as Pakistan to improve outcomes of febrile neutropenia in acute leukemia patients. The development of improved guidelines and practice of evidence-based antibiotic prescription in the region can aid in minimizing the existing care gap in the management of febrile neutropenia.
